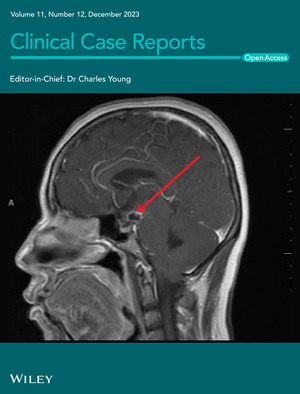# Cover Image

**DOI:** 10.1002/ccr3.8388

**Published:** 2023-12-25

**Authors:** Aliya F. Rehman, Alex F. Lazo‐Vasquez, Parjanya K. Bhatt, Tanya Quiroz, Joelle‐Ann Joseph, Sibel Gultekin, Nadine Montreuil, Candice A. Sternberg, Folusakin Ayoade

## Abstract

The cover image is based on the Case Report *Neurocysticercosis mimicking craniopharyngioma: A case report* by Aliya F. Rehman et al., https://doi.org/10.1002/ccr3.8166